# Interplay between the Localization and Kinetics of Phosphorylation in Flagellar Pole Development of the Bacterium *Caulobacter crescentus*

**DOI:** 10.1371/journal.pcbi.1002602

**Published:** 2012-08-02

**Authors:** Carolina Tropini, Kerwyn Casey Huang

**Affiliations:** 1 Biophysics Program, Stanford University, Stanford, California, United States of America; 2 Department of Bioengineering, Stanford University, Stanford, California, United States of America; Stockholm University, Sweden

## Abstract

Bacterial cells maintain sophisticated levels of intracellular organization that allow for signal amplification, response to stimuli, cell division, and many other critical processes. The mechanisms underlying localization and their contribution to fitness have been difficult to uncover, due to the often challenging task of creating mutants with systematically perturbed localization but normal enzymatic activity, and the lack of quantitative models through which to interpret subtle phenotypic changes. Focusing on the model bacterium *Caulobacter crescentus*, which generates two different types of daughter cells from an underlying asymmetric distribution of protein phosphorylation, we use mathematical modeling to investigate the contribution of the localization of histidine kinases to the establishment of cellular asymmetry and subsequent developmental outcomes. We use existing mutant phenotypes and fluorescence data to parameterize a reaction-diffusion model of the kinases PleC and DivJ and their cognate response regulator DivK. We then present a systematic computational analysis of the effects of changes in protein localization and abundance to determine whether PleC localization is required for correct developmental timing in *Caulobacter*. Our model predicts the developmental phenotypes of several localization mutants, and suggests that a novel strain with co-localization of PleC and DivJ could provide quantitative insight into the signaling threshold required for flagellar pole development. Our analysis indicates that normal development can be maintained through a wide range of localization phenotypes, and that developmental defects due to changes in PleC localization can be rescued by increased PleC expression. We also show that the system is remarkably robust to perturbation of the kinetic parameters, and while the localization of either PleC or DivJ is required for asymmetric development, the delocalization of one of these two components does not prevent flagellar pole development. We further find that allosteric regulation of PleC observed *in vitro* does not affect the predicted *in vivo* developmental phenotypes. Taken together, our model suggests that cells can tolerate perturbations to localization phenotypes, whose evolutionary origins may be connected with reducing protein expression or with decoupling pre- and post-division phenotypes.

## Author Summary

The development of multicellularity requires specialization and differentiation of individual cells. The process of differentiation requires the breaking of cellular symmetry, which can be achieved via asymmetric localization of proteins; cell division then gives rise to cells with different compositions and hence potentially different fates. However, little is known regarding the classes of changes in protein localization a cell can tolerate without disrupting development. *Caulobacter crescentus* is a model bacterium that, following cell division, gives rise to two differentiated daughter cells, only one of which is flagellated and motile. This process is regulated by two proteins, PleC and DivJ, located at opposite ends of the mother cell. Using computational modeling, we investigate the robustness of flagellar pole development to protein localization changes. Our analysis suggests that the development of *C. crescentus* is robust to a wide range of localization changes of PleC and DivJ. Furthermore, certain mutant localization patterns that would disrupt development can nevertheless support it when the mislocalized protein is present at higher abundance relative to wild-type. Our analysis highlights informative localization mutants that can be used to further deconstruct the requirements for *C. crescentus* development, and addresses the general requirements for protein localization in developmental processes and for engineering differentiation in biological systems.

## Introduction

The localization of proteins is highly regulated throughout all kingdoms of life. In eukaryotic cells, asymmetric distributions of proteins contribute to a diverse set of processes including cell-shape determination and motility [1], embryonic development [2], stem-cell maintenance [3], and the structural establishment of neurons and cilia [4]. Spatial organization is often dynamic, particularly throughout the cell cycle with the eventual generation of protein compositions that differ across the two halves of the cell. When this occurs, two distinct daughter cell types can be created by the post-cytokinesis segregation of the differential protein populations. The number of localized proteins in bacteria, and the biological processes in which they are engaged, are now known to be extensive; in the model bacterium *Caulobacter crescentus*, at least 10% of all proteins are non-uniformly localized and this subset covers all manners of function [5]. In many cases, localization is consistent with protein function, such as the coordination of cytokinesis by proteins that localize specifically to the division plane. Moreover, synthetic biology applications have begun to feature engineered systems utilizing protein localization to achieve specific functions such as increased metabolic pathway output [6]. However, relatively little is known regarding whether precise localization of the components of a complex system is *required* for achieving cellular functions, due to both the challenge of creating novel localization mutants and the absence of quantitative models for interpreting the mechanisms underlying changes in phenotype.

In *Caulobacter*, each round of the cell cycle involves an intricate program of spatially regulated developmental events that leads to the formation of two different cell types: a sessile, stalked cell and a motile, swarmer cell. During the cell cycle, the swarmer cell progresses through phases in which the flagellum is activated, shed, and replaced first with pili and then a stalk and holdfast. Development is controlled by several two-component signaling systems, which usually consist of a histidine kinase and its cognate response regulator. Histidine kinases can act either as a kinase or a phosphatase, to phosphorylate or dephosphorylate the response regulator, respectively [7]. Central to development is the master cell-cycle response regulator CtrA, which controls morphogenesis, DNA methylation, and many essential cell-cycle events [8], [9].

In recent work, we demonstrated that *Caulobacter* generates a spatial gradient of the active, phosphorylated form of CtrA that directly regulates DNA replication [10]. Employing a combination of mathematical modeling, single-cell microscopy, and genetic manipulation, we determined that this gradient is produced by asymmetric polar localization of the phosphorylation and dephosphorylation of CtrA by the bifunctional enzyme CckA. Our data indicated that cells robustly establish the asymmetric replicative fates of daughter cells before cell division effects physical compartmentalization. Importantly, localization of the phosphorylation or dephosphorylation activity alone is sufficient to establish and maintain the asymmetry.

The freely diffusing response regulator DivK regulates CtrA transcription and, with the polar-bound histidine kinases DivJ and PleC, controls the flagellar pole development (FPD) program in *Caulobacter*. DivJ plays a role in cell division and stalk development, and phosphorylates DivK *in vitro* and *in vivo* [11]–[13]. PleC is necessary for cell motility [14] and dephosphorylates DivK-phosphate [13], [15], [16]. Throughout this work, we will refer to the unphosphorylated and phosphorylated forms of DivK as K and 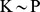, respectively.

**Figure 1:**
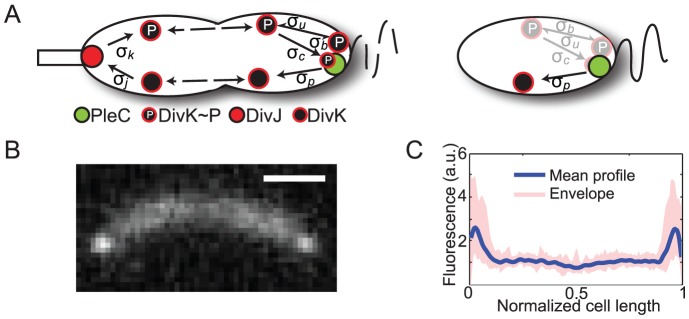
*Caulobacter crescentus* cells exhibit a non-uniform distribution of DivK-GFP. A) Ping-Pong model for the DivJ-DivK-PleC response regulator system in 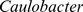 for a pre-divisional cell (left) and a swarmer cell (right). DivJ acts as a source of 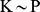 while PleC acts as a sink. High levels of 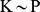 prevent flagellar pole development (FPD) in the pre-divisional cell. After cell division, only PleC is present in the swarmer cell, leading to dephosphorylation of 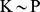 and subsequent FPD. B) Typical experimental DivK-GFP fluorescence profile in a wild-type *Caulobacter* cell. Scale bar is 1 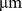. C) Quantification of the normalized DivK-GFP fluorescence intensity at the pole and midcell averaged over 25 cells. The shaded pink area represents the envelope of all the normalized fluorescence profiles.

Interestingly, DivJ and PleC are spatially regulated throughout the cell cycle [9], [13], [17]–[19]. At the time of cell division, DivJ is localized to the stalked pole while PleC is localized to the opposite swarmer pole (Fig. 1A) [13]. Cytokinesis leads to compartmentalization of PleC separate from DivJ, which is thought to dramatically reduce the levels of 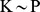 in the swarmer cell and permit FPD. DivK accumulates at both poles during growth, and the presence of swarmer pole-bound DivK alone is sufficient to inhibit FPD [8], [20]; DivK mutants that exhibit normal phosphorylation and dephosphorylation dynamics but impaired polar binding develop at the flagellar pole independent of cell division [8], [20].

The presence of the PleC phosphatase at the swarmer pole and the DivJ kinase at the stalked pole has motivated a “Ping-Pong” model for the passive dynamic polar localization of DivK in which the rapid diffusion of K and 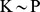 between the two poles maintains a steady-state level of 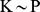 at the flagellar pole, thereby inhibiting FPD [20]. Adding to the complexity of this model is recent evidence that 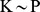 can act as an allosteric regulator to switch PleC from a phosphatase to an autokinase [21]. Although the dynamic regulation of DivK phosphorylation by DivJ and PleC is clearly affected by their localization at opposite poles, it remains unclear whether their polar localization is strictly necessary for FPD.

Previous genetic studies have indicated that a reduction in the levels of 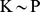 at the flagellar pole is a major requirement for FPD; non-phosphorylatable mutants of DivK do not localize to the swarmer pole [16], and in this case FPD takes place independent of cell division [20]. In wild-type cells, the asymmetric localization of DivJ and PleC at opposite poles means that their antagonistic kinase and phosphatase activities on DivK are separated by cytokinesis, leading to different developmental outcomes in the post-divisional stalked and swarmer cells. It has been suggested that localization of DivJ and PleC to opposite poles produces a pre-divisional gradient of 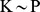 that helps to regulate FPD [22]. However, three lines of evidence argue that this simple model of 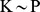 spatiotemporal dynamics is insufficient to account for the regulation of FPD. First, the phosphatase activity of PleC would actually create a minimum in the pre-divisional 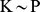 distribution at the swarmer pole, potentially permitting development pre-cytokinesis. Second, the mutant 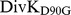 does not bind to the flagellar pole, yet it remains phosphorylatable and hence subject to potential gradient establishment by DivJ and PleC; nevertheless, cells expressing 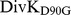 do not require cell division for FPD [8], [20]. Third, 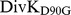 suppresses the defects of a *pleC*::Tn5 mutant, allowing FPD in the absence of PleC dephosphorylation, indicating that the phosphatase activity of PleC is not required for FPD despite its necessity for gradient formation [8], [20].

Taken together, these observations suggest that the progression of FPD is dictated primarily by the levels of 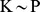 localized and bound to the flagellar pole, with higher levels inhibiting FPD. In the pre-divisional cell, high levels of 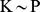 are maintained by DivJ, leading to enhanced polar binding and prevention of premature FPD. Cell division then segregates DivJ from the swarmer cell, leading to a reduction in both cellular and polar-bound 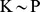 levels via PleC-mediated dephosphorylation and allowing FPD to progress in the swarmer cell (Fig. 1), while the stalked cell has increased 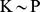 levels due to DivJ activity [20]. Although this model is qualitatively consistent with the developmental trajectory of wild-type cells, a quantitative framework is required to assay whether other strategies could lead to FPD independent of cytokinetic compartmentalization.

Here, we used experimental measurements of DivK fluorescence profiles to constrain potential models of K and 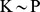 dynamics. Using this data, we developed and validated a reaction-diffusion model for the DivJ-DivK-PleC system. We used this model to make predictions regarding the developmental phenotypes of various localization mutants and discuss how these strains could provide insight into the importance of localization. We show that the localization of PleC and DivJ at opposite poles is likely to give rise only to very shallow gradients insufficient to produce asymmetry in response-regulator activity in the cytoplasm; instead, our simulations support the hypothesis that binding at the swarmer pole suffices to create functional polarity. Moreover, we show that in strains with stalked-pole mislocalization of PleC, the predicted lack of FPD can be rescued by over-expression of PleC, suggesting that development can be robust to changes in protein localization. Therefore, polarity may have evolved to counter the costs of high protein expression and to expand the phenotypic repertoire by dissociating the phenotypes of daughter cells.

## Results

### The molecular basis of DivK polar accumulation

In previous work, we used modeling to demonstrate that kinase and phosphatase kinetics are the dominant factors in the establishment of gradients of protein activity [10]. We found that spatial heterogeneities in other processes such as synthesis and degradation are unlikely to overcome rapid rates of diffusive mixing, or perturb the steepness of gradients formed by rapid kinase/phosphatase activity. Therefore, to analyze the distribution of 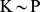 in the DivJ-DivK-PleC system, we focused on the biochemical kinetics of DivJ and PleC. Fluorescence loss in photobleaching experiments have established that DivK cycles between the poles within a 5-sec time scale, indicating rapid kinetics [20]. However, unlike the uniform fluorescence profile expected from a system relying purely on kinase and phosphatase activity, DivK-GFP levels increase at the poles [17]. These polar accumulations suggest that DivJ-mediated phosphorylation must actually be treated as a two-step process involving (i) binding of K to DivJ and (ii) subsequent release of 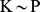. Furthermore, they also imply that phosphorylation kinetics are *slow* compared to the rate of binding, such that DivK spends a significant amount of time bound to DivJ at the pole; a similar conclusion applies for PleC-mediated dephosphorylation. Thus, we defined separate rate constants for binding and for catalysis (which we assumed is immediately followed by release): 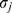 and 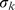 for binding and phosphorylation by DivJ, respectively, and 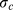 and 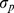 for binding and dephosphorylation by PleC, respectively. The magnitudes and spatial dependencies of these rates are regulated by the abundances and membrane distributions of DivJ and PleC and thus factor into the binding rates 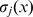 and 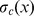; that is, 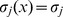 where DivJ is localized and 0 elsewhere, and 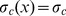 where PleC is localized and 0 elsewhere.

While polar accumulation may be achieved solely by direct binding of K or 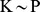 to DivJ and PleC, evidence that DivK-GFP accumulates at the swarmer pole even in the absence of PleC [20] suggests that DivK also binds to the swarmer pole in a PleC-independent manner that we will refer to as “polar binding.” To account for this behavior, we defined additional binding and unbinding rate constants (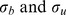, respectively) that are nonzero only at the swarmer pole. Given that the non-phosphorylatable mutant 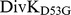 does not exhibit polar binding [16], we assumed that the DivK binds to the flagellar pole only in its phosphorylated state. Likewise, a 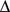 *divJ* mutant does not exhibit polar localization of DivK-GFP, indicating that DivJ-mediated phosphorylation is essential for DivK accumulation at the stalked pole [17].

Based on this evidence, we developed a minimal reaction-diffusion model for a pre-divisional cell considering two freely diffusing cytoplasmic species (K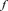 and 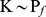), and three non-diffusing, membrane-bound species: K bound to DivJ prior to phosphorylation (K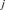), 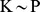 bound to PleC prior to dephosphorylation (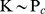), and 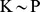 bound to the flagellar pole independent of PleC (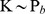). In the interest of keeping the number of parameters small, we assumed that the reverse reactions involving release of 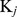 from DivJ without phosphorylation (and release of 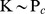 from PleC without dephosphorylation) are slow compared to the rates of phosphorylation and dephosphorylation, respectively, and therefore can be ignored. We modeled the distributions of these species along the length of a one-dimensional cell as shown schematically in Fig. 1A and represented mathematically as


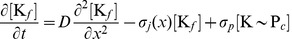
(1)


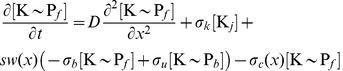
(2)


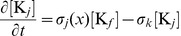
(3)


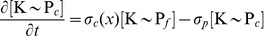
(4)


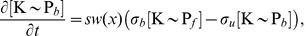
(5)

where 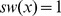 at the swarmer pole and 0 elsewhere. We assumed that diffusion is not affected by the phosphorylation state of DivK. Table 1 outlines the parameters that were used in all simulations.

**Table 1:** Details of kinetic parameters and localization functions in simulations of different strains.

Wild-type	Rates
wt	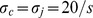 , 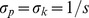 , 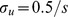 and 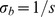 . 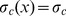 at the swarmer pole, and 0 elsewhere, 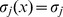 at the stalk pole and 0 elsewhere. In the PAR model, 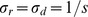 , 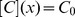 at the swarmer pole, 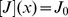 at the stalk pole and 0 elsewhere.

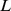
 is the cell length, 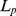 is the polar region length.

**Figure 2:**
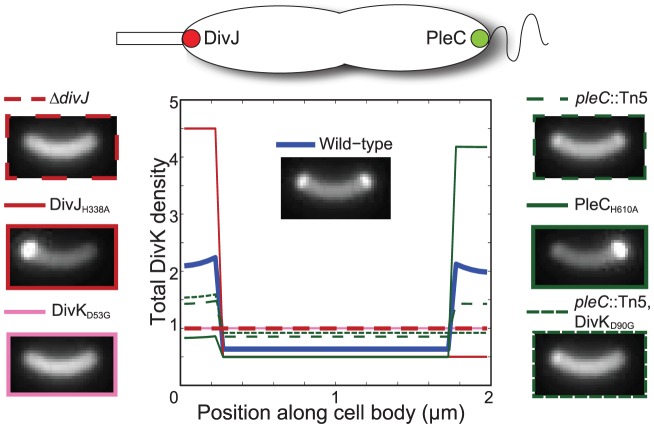
Mathematical modeling recapitulates experimental DivK-GFP distributions. Mathematical modeling of the spatial distribution of DivK in wild-type or mutant strains lacking DivJ (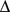 *divJ*) or PleC (*pleC*::Tn5) [17], with non-phosphorylatable DivK (DivK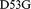) [16], a DivJ mutant lacking kinase activity (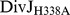) [16], a PleC mutant that is catalytically inactive (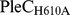) [16], or a DivK variant that does not bind to the pole (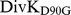) in a *pleC*::Tn5 background [20]. The sides and center inset show simulated microscopy data for the distributions shown in the middle plot computed numerically from our reaction-diffusion model. These distributions closely match the experimental microscopy data from the references given above. Wild-type rates: 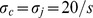, 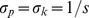, 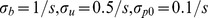.

Experimental measurements of the DivK-GFP distribution in wild-type and a variety of mutant 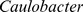 cells were used to estimate the magnitudes of the six unknown catalytic and binding rates in our model. To determine the relative amounts of DivK protein bound to different regions of the cell, we synchronized a wild-type population and quantified the polar and midcell concentrations of DivK-GFP in late pre-divisional cells. From Eq. 3, we expected that the steady-state ratio of polar, DivJ-bound K to freely diffusing K in the vicinity of the stalked pole to satisfy 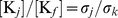, indicating that 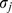 should be larger than 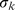 in order to observe polar accumulation. Although our fluorescence measurements could not distinguish between the two phosphorylation states K and 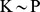, we estimated the pole-to-mid cell fluorescence ratio in wild-type cells as 
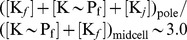
 (Fig. 1C). In simulations based on our model, we found that 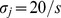 and 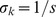 achieved a similar pole-to-midcell ratio of DivK protein (blue curve in Fig. 2).

Levels of DivK-GFP at the stalked and swarmer poles are roughly equivalent in pre-divisional cells; although it was not always possible to determine which was the stalked pole, a random ordering of the poles to determine the ratio gave a mean value of 0.9 and standard deviation 0.3 (Fig. 1B). Therefore, we set the parameters associated with PleC kinetics equal to those of DivJ (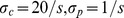). Furthermore, DivK-GFP levels at the swarmer pole of a *pleC*::Tn5 mutant are comparable to those in wild-type cells [17]. This observation required 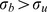 for significant levels of protein to be bound to the pole in the absence of PleC. For DivK molecules to be able to cycle quickly between the poles, we set 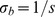 and 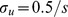. Given a diffusion constant 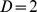 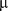
m^2^/s, this choice of parameters ensured that the vast majority of the DivK population in a cell would be bleached in less than 5 seconds by a laser focused on the stalked pole (Fig. S1), as was previously measured experimentally [20]. Since the rates of phosphorylation and dephosphorylation cannot be considerably faster than diffusion and still result in polar accumulation [10], we do not expect an appreciable midcell gradient of K and 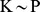. Indeed, the steady-state solutions to Eqs. 1–5 for both K and 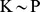 have a nearly flat midcell distribution, with a peak in the total DivK distribution at the poles due to DivJ, PleC, and flagellar-pole binding (blue curve in Fig. 2). The peak in DivK protein at the swarmer pole is due to increased levels of 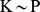, which would inhibit FPD in the pre-divisional cell.

To validate our model, we altered the PleC/DivJ spatial distributions and/or kinetics to mimic several previously characterized DivJ and PleC mutants (Fig. 2) [16], [17]. For each mutant, we used our computational prediction of the spatial distribution of DivK to simulate a typical DivK-GFP fluorescence image using the software package BlurLab (Methods), which generates simulated fluorescence microscopy data from 3D positions and intensities of fluorescent molecules [23]. In all cases, the predicted DivK-GFP distributions closely matched the corresponding experimental data (Fig. 2) [16], [17], [20]. Thus, our model successfully integrates the activities of PleC, DivJ and polar binding and provides a framework for evaluating the potential developmental consequences of changes in protein localization and expression.

### Model predictions for novel localization strains

Mathematical models provide a useful tool for predicting how genetic changes that affect localization and catalytic activity can be used to infer the spatial distribution of response regulator phosphorylation [10]. Here, we applied our model for the DivJ-DivK-PleC system to estimate the spatial distribution of 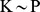 in strains with altered DivJ kinase or PleC phosphatase activity. We considered scenarios that reflect three potential phenotypes of mutations in DivJ or PleC: (1) concentrated at opposite poles (wild-type localization), (2) localized to the wrong pole (“mislocalized”), or (3) uniformly distributed throughout the cell membrane (“delocalized”). In addition to localization phenotypes, we also considered the effects of changes in expression levels, specifically to determine whether the predicted phenotype of a localization mutant could be altered by over-expression.

In each case, we mimicked a mutant phenotype by changing the spatial distribution and magnitude of 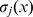 and 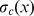 in Eqs. 1–5 (Table 1). We explored seven specific hypothetical strains: (1) wild-type, (2–4) PleC mislocalized to the stalked pole at 1×, 5×, or 25× wild-type expression levels, (5) delocalized PleC, (6) DivJ mislocalized to the swarmer pole, and (7) delocalized DivJ. All of these strains are experimentally realizable. PleC has been shown to delocalize in the presence of mutations in *podJ* [24], [25], and DivJ has been delocalized by removing 326 N-terminal residues that do not contain the catalytic domain [26]. Furthermore, the localization domain of DivJ could potentially be used to create a chimera with the DivJ stalked-pole localization sequence and the phosphatase catalytic domain of PleC that would constitute a mislocalized PleC; a similar strategy could be employed to construct a mislocalized DivJ.

**Figure 3:**
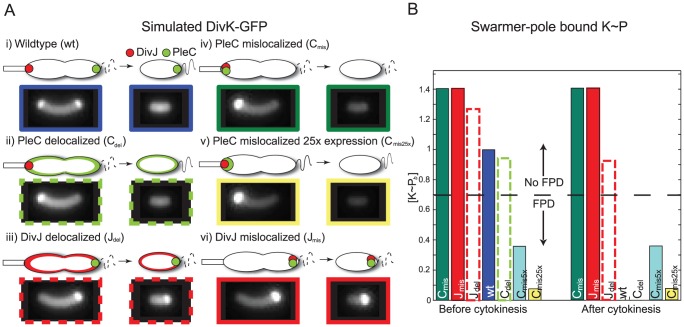
Wild-type and localization mutants of DivJ and PleC exhibit different levels of polar bound 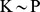 (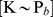). A) Simulated DivK-GFP fluorescence profiles for various localization and expression-level strains. B) Strains segregate into groups of high and low 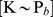 levels. The dotted line represents a putative level of 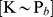 above which development is predicted to be inhibited. While we cannot determine the actual value of this threshold, a tunable system such as the mislocalized PleC strain would allow pinning down this value (or interval) more quantitatively.

Given that rapid kinetics are required to produce the wild-type fluorescence profile with significant polar accumulations of DivK-GFP, all species in our model reached their steady-state distributions on a time scale (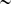
10 sec) much faster than the cell cycle. We therefore used our model to determine the steady-state distributions for each of the seven hypothetical localization mutants. We focused on both the expected distribution of DivK-GFP and the levels of polar-bound 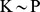, before cytokinesis in the pre-divisional cell and after in the swarmer cell. Simulated fluorescence profiles based on our numerical predictions (Fig. 3, Methods) can be directly compared with experimental measurements, while levels of 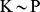 bound at the flagellar pole can be used to predict the developmental phenotype of pre- and post-divisional cells. Specifically, numerical solutions of our model for a given set of parameters can be used to determine whether a change in localization or expression will cause an increase or decrease in the level of polar-bound 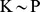 relative to wild-type, and hence whether a given strain should exhibit FPD independent of cytokinesis, only after cytokinesis, or not at all.

In some cases, simple physical considerations predict general trends in the 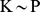 distributions across the various strains independent of our choice of parameters. For the wild-type phenotype, cell division separates the 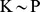 source (DivJ) and the sink (PleC), eliminating the polar accumulation of 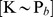 and thereby allowing FPD (Figs. 3, S2) [22]. When PleC is mislocalized, PleC and DivJ compete to determine the phosphorylated fraction of DivK at the stalked pole, and the rest of the cell maintains constant levels of K and 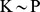 due to diffusion (Fig. S2). 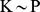 levels can thus be modulated by the expression level of mislocalized PleC, with lower 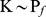 and 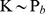 levels for higher PleC levels (Fig. S2). After cytokinesis, the uniform midcell distribution of 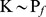 remains unchanged in both daughter cells since DivJ and PleC are not separately compartmentalized (Fig. S2). Thus, when PleC is mislocalized, we expect the FPD phenotype to be unaffected by cytokinesis, and sufficiently high levels of PleC expression should reduce the 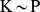 levels at the swarmer pole enough to initiate premature FPD prior to cytokinesis. Mislocalization of DivJ has a similar behavior with uniform levels of K and 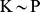 throughout the cytoplasm (Fig. S2).

Delocalization of PleC alters the level of polar-bound 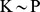 in the pre-divisional cell, but upon cytokinesis the swarmer cell again has PleC without DivJ, which should eliminate the polar 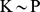 and allow FPD to progress similar to the wild-type case (Figs. 3, S2). In contrast, delocalization of DivJ increases the levels of 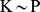 at the swarmer pole, since the source of DivK phosphorylation has been shifted closer to the swarmer pole. For this strain, we also predict a significant reduction in the total levels of DivK-GFP at the stalked pole, resulting in a more uniform cytoplasmic distribution. After cytokinesis, the swarmer cell has both a sink and a source of 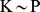, and hence the level of polar-bound 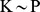 should depend on the relative catalytic activities of DivJ and PleC.

Using numerical simulations of our reaction-diffusion model, we quantified the overall DivK distribution and the polar-bound 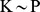 concentration (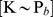) in each mutant. Figure 3 shows simulated DivK-GFP fluorescence intensities for the strains described above (Methods); details and quantification of the respective concentration distributions can be found in the Supplemental Information (Fig. S2). Even without experimental quantification of the total DivK protein levels, we can interpret the 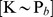 levels relative to wild-type, with higher levels more unlikely to exhibit FPD. Based on the levels of 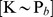 before and after cytokinesis (Fig. 3B), we predict that in the pre-divisional cell, only PleC mislocalized and over-expressed by 5× (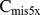) and 25× (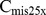) are likely to exhibit FPD, while the post-divisional wild-type (wt) and delocalized PleC (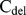) cells will develop normally (Fig. 3B).

### Relative phosphorylation levels are independent of parameters

Although the parameters we used in our model were motivated by experimental data (Figs. 1,2), we were interested to determine whether the predicted ordering of 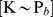 levels across our hypothesized localization/expression strains (Fig. 3B), and therefore the predicted FPD phenotypes, would be robust to changes in the kinetic parameters. To do so, we perturbed the rates (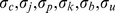) over a wide physiological range and numerically determined the steady-state levels of 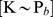 pre- and post-division. For a subset of the strains, the relative ordering of 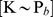 could be readily inferred from physical considerations (Figs. S2, S3). For instance, we predict that a strain with PleC over-expressed 25× will always have lower 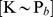 than a wild-type cell: over-expressing the sink will deplete the cell of 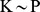, and thus the cell will always be more likely to initiate FPD, independent of cytokinesis.

However, the relative 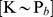 ordering for many strains is nontrivial due to the competition among DivJ, PleC, polar binding, and diffusion; hence our mathematical model was critical for quantitatively analyzing the system kinetics. We explored the parameter space by scaling each rate constant by a random factor selected logarithmically between 0.25 and 4 and calculated the levels of 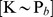 for all hypothesized localization/expression phenotypes, before and after cytokinesis. Although the absolute levels of 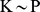 varied substantially due to changes in the overall kinase or phosphatase rates, the specific ordering of polar-bound 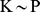 levels in pre-divisional cells was identical to that in Fig. 3B for each of 10,000 random parameter sets (Fig. S3). Therefore, given that wild-type cells do not exhibit FPD when cytokinesis is blocked [20], we predict that all hypothesized strains with higher than wild-type levels of 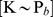 (delocalized DivJ, 1× mislocalized PleC, and mislocalized DivJ) will also not undergo FPD before the completion of cytokinesis.

Furthermore, simulations of the delocalized PleC mutant resulted in 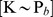 levels very close to wild-type (for this strain, 50% of the parameter sets yielded 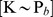 levels within 10% of wild-type), and thus we also predict that these cells will not undergo FPD pre-cytokinesis. On the other hand, we predict that sufficiently over-expressed, mislocalized PleC strains will have 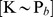 at a low enough level to allow FPD pre-division. For 99.98% of the parameters sets, the 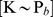 levels in the mutants with PleC mislocalized and over-expressed 25× harbor 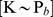 levels less than 25% of wild-type.

The ordering of 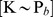 levels in simulations of the swarmer cell after compartmentalization is even more pronounced, with wild-type and delocalized PleC strains exhibiting the lowest levels, followed by mutants with 25× and 5× mislocalized PleC. We predict that cells with delocalized DivJ will have higher 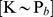 levels than all strains but mislocalized DivJ or PleC. As in pre-divisional cells, 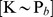 levels in the mislocalized PleC and mislocalized DivJ mutants have a flat 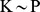 distribution between the poles that is unaffected by division.

Given that wild-type cells initiate FPD after cytokinesis, we predict that mutants with delocalized PleC will develop normally. Mutants with over-expressed, mislocalized PleC should show the same developmental phenotype in the absence of cytokinesis, and the extremely low levels of polar-bound 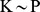 in cells with 25× over-expression of mislocalized PleC suggest that such a strain should exhibit FPD independent of cytokinesis. However, the levels of 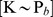 for mutants with either delocalized DivJ or normal expression levels of mislocalized DivJ/PleC are not significantly reduced by cytokinesis to levels lower than pre-divisional wild-type cells, and hence we predict that these mutants will not exhibit FPD.

**Figure 4:**
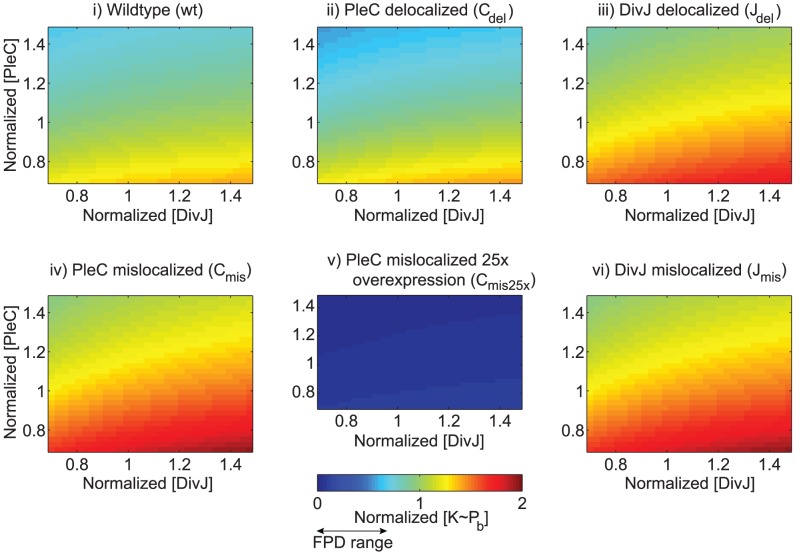
PleC and DivJ gene expression noise have little effect on FPD. Simulated 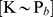 levels normalized to wild-type levels for various localization and expression-level strains as the magnitude of 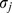 and 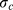 are varied. The only strain that is likely to have altered FPD phenotype due to noise is the PleC delocalized mutant, which, if PleC levels are high, could have low enough 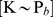 levels to undergo development pre-cytokinesis.

To mimic the effects of PleC and DivJ gene expression noise on our predicted FPD phenotypes, we varied 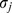 and 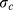 between 0.7 and 1.5 times the values used in Fig. 3 and plotted 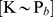 relative to the wild-type levels in the pre-divisional cell before cytokinesis (Fig. 4). The only strain whose phenotype is likely to be affected by noise is the PleC delocalized mutant, which, if PleC levels are high and DivJ levels are low, could have low enough 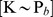 levels to undergo development pre-cytokinesis. Experimentally, this effect may manifest experimentally as a range of developmental phenotypes in cells from this mutant strain.

Taken together, our simulations predict a systematic ordering of 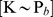 levels that is independent of the choice of parameters. This ordering corresponds to predictions of the developmental outcomes for many of the strains considered here. Moreover, our simulations indicate that the creation of a mutant with mislocalized PleC under inducible expression would provide insight into the quantitative relationship between the polar-bound 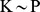 concentration and FPD progression by revealing the largest concentration 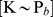 that still allows FPD. In contrast, we predict that the other strains have levels of 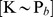 that lie at more extreme ends of the developmental spectrum, indicating a more clearcut FPD phenotype.

### PleC allosteric regulation

**Figure 5:**
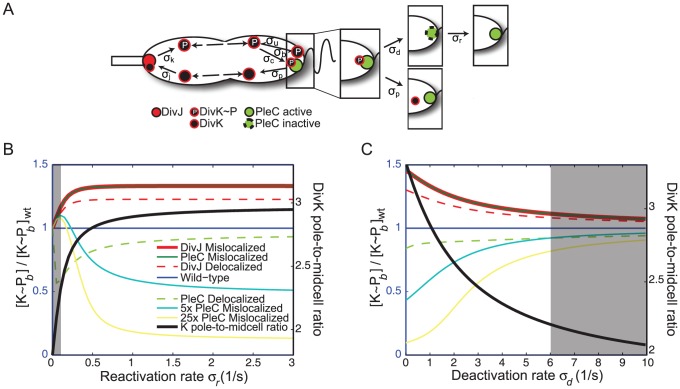
The PAR model incorporating allosteric regulation of PleC by 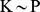 predicts similar FPD phenotypes to the Ping-Pong model. A) In addition to the reactions occurring in the Ping-Pong model, *in vitro* data suggests that 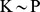 can also cause PleC to switch from a phosphatase into an autokinase, incapable of dephosphorylating 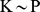. We assume that the deactivation of PleC as a phosphatase occurs with rate 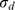 when 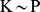 is bound, and that PleC spontaneously reactivates with rate 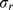. B) 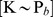 levels relative to wild-type vary significantly only for small 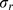, in which regime the pole-to-midcell ratio does not match experimental data. C) 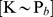 levels relative to wild-type converge at large 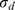; in this regime most of the available PleC is inactive again and the model does not reproduce the experimentally observed DivK pole-to-midcell ratio. The gray shaded areas in B) and C) represent the regimes in which the pole-to-midcell ratio is more than one standard deviation away from the experimentally observed ratio of 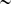
3.

Recent biochemical evidence has suggested that PleC is bifunctional, with 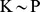 acting as an allosteric regulator to switch PleC from a phosphatase to an autokinase incapable of dephosphorylating 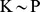 [21]. To investigate whether this additional regulation is likely to play a role in controlling FPD *in vivo*, we modified our model to include an additional loop in which a complex of 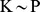 and PleC can result in the switching of PleC into an autokinase with deactivation rate 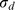. To ensure that a small number of 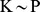 molecules cannot switch the entire PleC population into autokinases, this deactivated form must reactivate to a phosphatase, which we assumed occurs spontaneously with rate 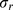 (Fig. 5A, Table 1).

Our modified model (the “PAR” model) thus considers the subdivision of the PleC population (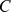) into three species: unbound PleC in its active (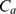) and deactivated (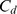) forms, and active PleC bound to 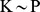 (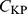). DivJ is assumed to always be active as a kinase and is represented by 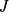. Whereas in Eqs. 1–5 the expression levels of PleC and DivJ were incorporated into the rate constants, we now explicitly consider the concentrations of PleC and DivJ such that all rate constants represent intrinsic properties of the respective intermolecular interactions (Fig. 5A):


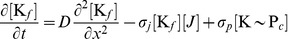
(6)


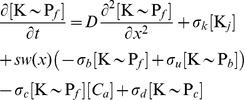
(7)


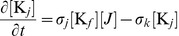
(8)


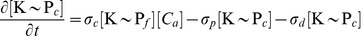
(9)


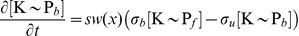
(10)


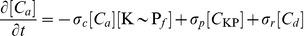
(11)


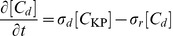
(12)


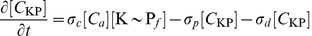
(13)

When PleC and DivJ levels are comparable to or greater than DivK levels, the system behaves similarly to the non-allosteric model (compare Figs. S2 and S4), since there is always a pool of active PleC to dephosphorylate 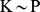. In contrast, when PleC levels are very low, the PleC kinetics are similar to those of a saturated enzyme. Importantly, one effect of decreasing the PleC and DivJ concentrations relative to DivK is the reduction of polar accumulation: PleC concentrations of less than 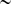
20% of the total DivK concentration lead to pole-to-midcell ratios less than our experimentally observed values (Fig. S5). Therefore, to maintain our experimentally observed pole-to-midcell ratio of DivK-GFP, we hereafter study the regime in which overall PleC and DivJ levels are similar to DivK levels. In this regime, our PAR reaction-diffusion model with with 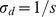 and 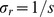 again reproduces the experimental fluorescence data for the strains described in Fig. 2; in particular, our model shows the expected polar accumulation of DivK-GFP relative to midcell in a wild-type cell (Fig. S6).

Next, we explored how the PleC deactivation and reactivation rates affect the accumulation of 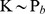 at the pole. Varying the reactivation rate in the PAR model did not correlate significantly with changes in the wild-type concentration profile (Fig. S7A); in contrast, increasing the deactivation rate lowered the total amount of 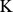 and 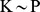 bound to the poles (Fig. S7B). As more PleC became inactive, less 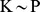 was dephosphorylated, decreasing 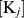; as the fraction of PleC that was available to bind 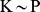 dropped, 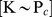 decreased until all PleC ended up inactivated by the large 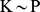 pool (Fig. S7). Such high 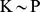 levels combined with low polar accumulations 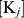 and 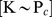 are not consistent with experimental evidence from the fluorescence experiments previously described (Fig. 2), justifying our selection of the deactivation and reactivation rates at intermediate values of 1/s. In this regime the DivK pole-to-midcell ratio is close to 3 (Fig. 5B,C), as we observed experimentally (Fig. 1C). A higher reactivation rate would not affect the polar ratio; however, in the limit of very high 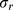 the PAR model is reduced to the Ping-Pong model, as all of the PleC pool would be active, equivalent to having large levels of PleC compared to DivK.

Similar to our analysis of the Ping-Pong model, we ordered the 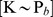 levels of each strain in the PAR model to infer the FPD phenotype (Fig. S8). In the PAR model, the ordering was slightly changed compared to the Ping-Pong model, but our overall conclusions were not affected (Figs. S8, S9). We still expect the DivJ delocalized, PleC mislocalized, and DivJ mislocalized mutants to not exhibit FPD either before or after cytokinesis. In contrast to the Ping-Pong model, the PleC delocalized mutant could undergo FPD prior to cytokinesis if the PleC reactivation rate is very fast (Fig. S10). This scenario causes more PleC to participate in a futile deactivation/reactivation cycle without dephosphorylating DivK, leading to higher overall 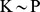 levels in all mutants. However, the PleC delocalized mutant have the least relative increase in 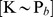 upon a reactivation rate increase among all the strains, since this effect is spread out over the cell instead of being concentrated at the swarmer pole. The opposite effect is predicted to occur when the reactivation rate decreases in the PleC mislocalized and over-expressed mutants, whose 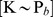 levels increase relative to wild-type.

In the post-divisional swarmer cell, the relative 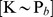 ordering is very similar to the non-allosteric, Ping-Pong model (compare Figs. S3 and S9). In the PAR model, we also predict FPD in the PleC delocalized mutant and in the the 25× and 5× PleC mislocalized mutants. Overall, 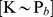 ordering is the same as the Ping-Pong model, except that the PleC mislocalized and over-expressed mutants have increased 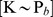 levels relative to the wild-type as the reactivation rate decreases, which causes the DivJ delocalized mutant to appear lower in the ordering. Similar to the Ping-Pong model, the DivJ delocalized, PleC mislocalized, and DivJ mislocalized mutants did not experience a large reduction in 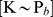 levels after cell division, and hence these strains are not expected to undergo FPD. Our simulations predict that PleC allosteric regulation should have little effect on most conclusions of the Ping-Pong model, with the possible exception of a delocalized PleC mutant that should be informative about the strength of PleC regulation by 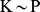 *in vivo*.

## Discussion

Sub-cellular localization of histidine kinases in bacteria can give rise to asymmetries in response regulator activities, thereby creating the basis for differential developmental outcomes [10]. Here, we have developed a reaction-diffusion model that predicts that the distributions of the phosphorylated and unphosphorylated DivK species are relatively homogeneous in the pre-divisional cytoplasm of *Caulobacter* cells. 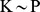 accumulation at the swarmer pole allows for tight prevention of FPD and this inhibition is lost once the cell divides and the phosphatase PleC is left alone to dephosphorylate 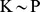. A gradient is not required in this system; instead, the asymmetric localization of the kinase DivJ and phosphatase PleC promotes a switch-like behavior. Importantly, although division enhances the asymmetry to ensure switch-like dephosphorylation of 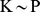, if the levels of 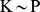 are kept low enough by over-expression of PleC, we predict that division is not required to activate FPD.

Our computational model reproduces experimental DivK-GFP fluorescence data from seven DivJ, DivK, and PleC mutant strains with a single set of binding and enzymatic rates (Fig. 2). Given this validation, we applied our model to predict 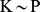 levels for several mutant localization phenotypes. We have shown that our predictions are independent of the choice of rates, indicating that our model makes general predictions regarding the developmental phenotype of each mutant strain.

Comparison of the Ping-Pong and PAR models of DivK steady-state levels revealed that the behavior of the DivJ-DivK-PleC system is not significantly affected by the allosteric regulation of PleC as long as PleC deactivation does not dominate the system dynamics. If the PleC deactivation rate is very high (or conversely, if the PleC reactivation is very low), only the PleC delocalized mutant is significantly affected relative to wild-type in the pre-divisional cell (Figs. S7, S9). In general, all strains are expected to lack FPD in the pre-divisional cell except for mutants with 25× and 5× over-expressed and mislocalized PleC. On the other hand, in the post-divisional swarmer cell, all strains are expected to develop at the flagellar pole except the PleC mislocalized, DivJ mislocalized, and DivJ delocalized strains. The observation that random variations in model parameters do not affect our conclusions (Fig. 4) indicates that the DivJ-DivK-PleC system is robust to noise and fluctuations in catalytic rates and expression levels. It is possible that allosteric regulation of PleC is required for other downstream reactions during development. In this case, our work then reveals that experiments focusing only on DivK regulation are insufficient to fully explore the origins of PleC localization and regulation.

**Figure 6:**
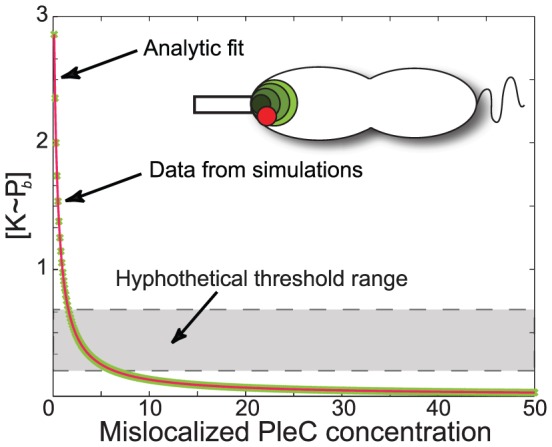
Over-expression can compensate for mislocalization to restore flagellar pole development. Polar-bound 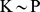 in a PleC-mislocalized cell as a function of the concentration of PleC (green circles in inset; DivJ is represented as a red circle). The grey shaded area shows a hypothetical range of thresholds separating FPD phenotypes similar to Fig. 3B: above the grey box we predict that flagellar pole development (FPD) will be inhibited while below the grey box FPD will proceed in both pre-divisional and swarmer cells. The data fits well to an inverse relationship 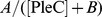 shown as a red solid line (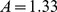, 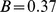).

Our analysis also suggests experiments that would illuminate the mechanism underlying regulation of FPD. Tunable expression of mislocalized PleC would allow the characterization of the 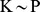 levels required to switch between development and non-development. We predict that this mutant should not change developmental state upon division, and by titrating the induction levels it should be possible to determine the expression threshold for FPD, with the flagellar-pole-localized levels of 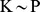 varying inversely with expression levels of stalked-pole-mislocalized PleC (Fig. 6). This result also highlights the non-trivial complementarity of localization and expression levels in this system, via the novel prediction that the phenotype caused by mislocalization of PleC can be rescued by its over-expression.

Our study reinforces the importance of mathematical modeling for deconstructing complex biological networks, and raises questions regarding the importance of the simultaneous localization of DivJ and PleC, particularly in light of a potential gene duplication event that may have led to the specialization of these two histidine kinases with 45% amino acid sequence similarity [27], [28]. Moreover, the robustness of FPD to changes in localization and expression levels would allow the cell to alter kinase localization to address other functions such as stalked-pole development without disrupting swarmer pole events. Given that distributions of 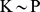 similar to wild-type can be achieved when either PleC or DivJ are delocalized (Fig. 3, S2), their polar accumulation may indicate that other downstream events and reactions require a high local concentration of these kinases for stalked-pole development; in this study, we have investigated only one aspect of development and PleC and DivJ are known to participate in multiple developmental events. Finally, our simulations suggest that synthetic biological systems with cellular asymmetry could be constructed without requiring the full complexity of their natural counterparts.

## Methods

### Growth conditions and imaging

*C. crescentus* CJ403 expressing DivK-GFP was grown as described previously [29]. Cells were synchronized using Percoll density centrifugation [30]. Synchronized swarmer cells were resuspended in peptone-yeast extract (PYE) medium to an 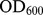 of 0.2–0.3 and imaged on 1% agarose pads. Imaging was performed on a Nikon Eclipse Ti-E inverted microscope with a Nikon Plan Apo 100× objective (numerical aperture of 1.4) running 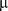
Manager. Cell boundaries and fluorescence linescans along the longitudinal axis of the cell were determined using MicrobeTracker [31]. In Fig. 1C, for each cell we computed the average fluorescence excluding the poles, and normalized the intensity profile to this mid-cell average. After normalizing position along the midline to the cell length, we computed the mean fluorescence profile along the normalized coordinate from 0 to 1. The variability was defined as the maximum and minimum normalized intensity at each position along the cell midline.

### Computational modeling

In order to estimate the rate of DivK diffusion, we used Monte Carlo simulations to calculate the minimum diffusion constant required to ensure that a given percentage of a uniformly distributed population would be photo-bleached within 5 seconds, where a particle is considered bleached if it approaches within 300 nm of the left pole of the cell (Fig. S1). For 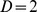 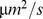, 99% of the molecules would be photo-bleached within 5 seconds, similar to experimental observations for DivK-GFP [20]; this rate compares favorably with the experimentally measured diffusion constant of a maltose-binding protein in *Escherichia coli* [32]. Increasing 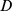 increases the rate at which K and 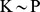 find their binding partners, thereby increasing the fraction of DivK bound to PleC, DivJ, and to the pole. In the various localization strains, this scenario resulted in increased 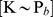, particularly for the DivJ delocalized, wild-type, and PleC delocalized strains, but the 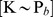 ordering was maintained. The mislocalized mutants were not affected, since the 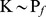 distribution is flat and hence is not sensitive to changes in 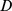.

Unless otherwise noted, we use 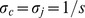, 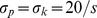, 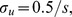 and 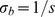 (Table 1). In Fig. 2, to simulate the non-catalytic interaction of K and 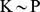 with DivJ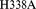 and PleC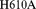, we set the catalytic rates 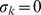/s and 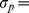
0/s, respectively. K and 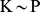 are then released after binding with rate 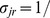
s and 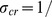
s without change in their phosphorylation state. In the PAR model, we include 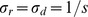 (Table 1). Furthermore, given that DivK accumulates at both poles in the absence of PleC [17], we infer the activity of a background phosphatase with rate 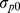. We exclude the possibility of a significant background kinase since a DivJ mutant that has no catalytic activity (DivJ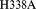) does not exhibit DivK-GFP accumulation at the swarmer pole [16]. We have previously shown that background kinase and phosphatase activity does not significantly affect response regulator distributions [10]. In Figs. S5, S7, S10 the simulations were carried out varying only the concentrations of DivK (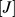) and PleC (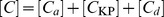), 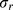 and 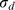. Table 1 provides the kinetic parameters and localization profiles in the simulations of each strain.

### Simulated microscopy

We used the software package BlurLab [23] to generate simulated microscopy images of DivK-GFP from 1D computational distributions. To create the 3D positions of fluorescent molecules within a pre-divisional cell, we modeled a crescent-shaped cell as a bent cylinder 3.5 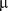
m in length and 0.5 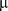
m in diameter with a radius of curvature of 1.5 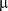
m and hemispherical poles, for a total length of 4 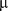
m. Swarmer cells were modeled as a bent cylinder 1.1 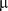
m in length and 0.5 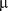
m in diameter with a radius of curvature of 1.5 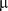
m and hemispherical poles for a total length of 1.6 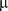
m. Within these volumes we positioned 10,000 molecules in the pre-divisional cell (4,000 in the swarmer cell) so that their spatial distribution matched the 1D concentration profiles from our computational modeling. The coordinates were then used by BlurLab to compute the expected fluorescence distribution utilizing a point spread function for a 100× objective with numerical aperture 1.4.

## Supporting Information

Figure S1
**Monte Carlo simulations indicate that the amount of unbleached DivK-GFP after 5 seconds of photobleaching at one pole should decrease with increasing diffusion constant** ***D*** **.** When 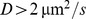, the unbleached fraction is 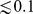
%.(EPS)

Figure S2
**Simulated DivK concentration profiles using the Ping-Pong model for wild-type cells and localization mutants of DivJ and PleC.** Steady-state distributions for total DivK (left), 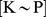 (center), and 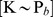 (right) are plotted for a pre-divisional cell (A) and post-divisional swarmer cell (B) for the set of localization and expression strains described in the main text and Fig. 3. The pre-divisional cell is assumed to be 2 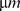 in length, with a 60/40 split between stalked and swarmer compartments post-cytokinesis. Simulated fluorescence profiles of the total DivK distributions can be found in Fig. 3.(EPS)

Figure S3
**Relative ordering of** 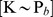 **levels for the steady-state solutions of the Ping-Pong model among PleC and DivJ localization and expression level strains in a pre-divisional (A) and swarmer (B) cell.** Colors indicate different strains, and are the same as in Fig. S2.(EPS)

Figure S4
**Simulated DivK concentration profiles using the PAR model for wild-type and localization mutants of DivJ and PleC.** Steady-state distributions for total DivK (left), 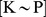 (center), and 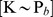 (right) are plotted for a pre-divisional cell (A) and post-divisional swarmer cell (B) for the set of localization and expression strains described in the main text and Fig. 3. The pre-divisional cell is assumed to be 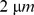 in length, with a 60/40 split between stalked and swarmer compartments post-cytokinesis. Simulated fluorescence profiles of the total DivK distributions can be found in Fig. S8.(EPS)

Figure S5
**The total DivK swarmer pole-to-midcell ratio increases with increasing PleC concentration and saturates when [PleC]** 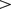 **[DivK].** Steady-state solutions to the PAR model were determined for DivJ concentration equal to that of PleC. The gray shaded area represents the region in which the pole-to-midcell ratio is more than one standard deviation away from the experimentally observed ratio of 3.(EPS)

Figure S6
**Mathematical model including allosteric regulation of PleC recapitulates experimental DivK-GFP distributions.** Mathematical modeling of the spatial distribution of DivK in wild-type or mutant strains lacking DivJ (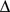 *divJ*) or PleC activity (*pleC*::Tn5) [17], with non-phosphorylatable DivK (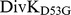) [16], a DivJ mutant lacking kinase activity (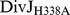) [16], a PleC mutant that is catalytically inactive (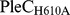) [16], or a DivK variant that does not bind to the pole (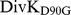) in a *pleC*::Tn5 background [20]. The sides and center insets show simulated microscopy data for the distributions shown in the middle plot. These distributions match the experimental microscopy data from the references indicated above. Wild-type rates: 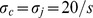, 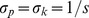, 
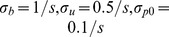
,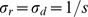.(EPS)

Figure S7
**The DivK pole-to-midcell ratio varies over a broad range for all values of the PleC reactivation rate (A) but decreases with increasing deactivation rate (B).** Each data point represents a steady-state solution to the PAR model for a pair of randomly chosen values of 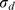 and 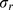 between 0 and 100; all other rates were kept fixed at the values used in Fig. 3.(EPS)

Figure S8
**The PAR model of wild-type and localization mutants of DivJ and PleC reveals the same ordering of polar bound** 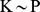 **(** 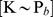 **) as the Ping-Pong model.** A) Simulated DivK-GFP fluorescence profiles for various localization and expression-level strains. B) Strains segregate into groups of high and low 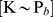 levels. The dotted line represents a putative level of 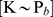 above which development is predicted to be inhibited.(EPS)

Figure S9
**Relative ordering of** 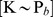 **levels for the steady-state solutions of the PAR model among PleC and DivJ localization and expression level strains in a pre-divisional (A) and swarmer (B) cell.** Colors indicate different strains, and are the same as in Fig. S4.(EPS)

Figure S10
**The amount of K** 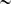 **P bound to the flagellar pole in the PleC delocalized mutant relative to the wild-type increases with increasing reactivation rate but is always less than 1.** The steady-state solutions were computed using the PleC allosteric regulation model in Eqs. 6–13.(EPS)
